# Performance sensitivity analysis of brain metastasis stereotactic radiosurgery outcome prediction using MRI radiomics

**DOI:** 10.1038/s41598-022-25389-7

**Published:** 2022-12-05

**Authors:** David A. DeVries, Frank Lagerwaard, Jaap Zindler, Timothy Pok Chi Yeung, George Rodrigues, George Hajdok, Aaron D. Ward

**Affiliations:** 1 Department of Medical Biophysics, Western University, London, N6A 3K7 Canada; 2 Gerald C. Baines Centre, London Health Sciences Centre, London, N6A 5W9 Canada; 3 Department of Radiation Oncology, Amsterdam University Medical Centre, Amsterdam, 1081 The Netherlands; 4 Department of Radiation Oncology, Haaglanden Medical Centre, Den Hague, 2512VA The Netherlands; 5 Holland Proton Centre, Delft, 2629JA The Netherlands; 6 RefleXion Medical, Hayward, 94545 USA; 7 Department of Oncology, Western University, London, N6A 3K7 Canada

**Keywords:** Cancer imaging, Radiotherapy, CNS cancer, Software, Biomedical engineering

## Abstract

Recent studies have used T1w contrast-enhanced (T1w-CE) magnetic resonance imaging (MRI) radiomic features and machine learning to predict post-stereotactic radiosurgery (SRS) brain metastasis (BM) progression, but have not examined the effects of combining clinical and radiomic features, BM primary cancer, BM volume effects, and using multiple scanner models. To investigate these effects, a dataset of n = 123 BMs from 99 SRS patients with 12 clinical features, 107 pre-treatment T1w-CE radiomic features, and BM progression determined by follow-up MRI was used with a random decision forest model and 250 bootstrapped repetitions. Repeat experiments assessed the relative accuracy across primary cancer sites, BM volume groups, and scanner model pairings. Correction for accuracy imbalances across volume groups was investigated by removing volume-correlated features. We found that using clinical and radiomic features together produced the most accurate model with a bootstrap-corrected area under the receiver operating characteristic curve of 0.77. Accuracy also varied by primary cancer site, BM volume, and scanner model pairings. The effect of BM volume was eliminated by removing features at a volume-correlation coefficient threshold of 0.25. These results show that feature type, primary cancer, volume, and scanner model are all critical factors in the accuracy of radiomics-based prognostic models for BM SRS that must be characterised and controlled for before clinical translation.

## Introduction

Primary cancers can spread to form brain metastases (BMs). Improved treatments have lengthened patient survival, leading to BM incidence in 20–40% of patients^1^. Since patients typically have advanced cancer extracranially when BMs develop, BMs are less likely to cause death, but do cause serious symptoms affecting quality of life^2^. Prognosis is poor due to extracranial factors, with a median survival of 7 months^3^. It is therefore critical to choose the optimal treatment for BMs at the outset, as there is limited time to pivot to an alternative treatment approach.

Common treatments include surgical resection and radiation therapy, along with newly developed systemic targeted and immunotherapy agents. Surgery is an effective option, but is contraindicated by inoperable BMs near eloquent locations and patient comorbidities. Radiation-based treatments offer a non-invasive, but effective alternative^4^. Whole-brain radiation therapy irradiates the entire brain over many fractions with the intent to spare healthy tissue. Stereotactic radiosurgery (SRS) uses highly conformal radiation doses delivered in 1–3 fractions to spare healthy tissue and cognitive function^4^. SRS may be delivered on a linear accelerator (linac), or on more specialized machines, such as Gamma Knife or Cyber Knife. SRS can also be hypofractionated to 5 fractions which will be defined as stereotactic radiotherapy (SRT).

Despite the advantages of SRS, it is not always successful; SRS fails for up to 30% of BMs^5^. In these cases, patients may continue to experience decreased quality of life from the BM. SRS also has side-effects^4^ including possible radiation necrosis which may increase patient steroid dependency.

It would be advantageous to have a prognostic model predicting whether a BM would progress after SRS. Such a model could aid in justifying SRS dose escalation or deciding between surgery and SRS. While prognostic models for BM patient overall survival have been developed, these do not inform if individual BMs will respond to SRS^6,7^. Previously reported prognostic factors for BM progression post-SRS include BM volume, dose and fractionation, and BM appearance in pre-treatment T1-weighted contrast-enhance magnetic resonance imaging (T1w-CE MRI)^2,7,8^.

Previous studies linking BM MRI appearance to SRS outcomes distilled all the MRI information into a single, qualitative appearance score, such as homogeneous, heterogeneous, or rim-like enhancement. Machine learning (ML) models and radiomic analysis of MRI have been incorporated into many health fields to provide prognostics^9–11^. Radiomics provides quantitative image analysis by extracting computational features from voxel data. These features can be combined using ML techniques to predict a desired outcome.

Previous studies have reported on prediction of SRS outcomes for BMs treated with Gamma Knife or Cyber Knife. They investigated the use of radiomic features from multiple MRI sequences, the planned SRS dose distribution, and the peri-tumoural region beyond the treated BM^12–16^. There have also been similar studies for BM SRT and post-surgical resection SRS using Gamma Knife^17–19^.

While these studies have offered preliminary insights into BM SRS prognostics, there remains an unmet need for a study that provides adequate evidence supporting the pursuit of external, multi-center validation of a model that predicts the outcome of BM SRS. To date, ML studies in this area have not examined the effects of primary cancer type, BM volume, and multiple MR scanner models on predictive accuracy. Investigating the effect of primary cancer site is important to consider as current overall survival prognostic models have been specifically developed for individual cancer sites^6^. Furthermore, some current ML studies use patient samples with mixed primary cancer sites, while some ML studies use single cancer site samples, with possible differences between these approaches remaining unexplored^14,15^. Exploring the BM volume and MR scanner effects is also important as both region-of-interest volume and MR scanner model have widely been found to have effects on the performance of radiomics systems^20–23^. There has also not yet been a robust comparison of the accuracy of clinical and radiomic features for this task. Our study addresses this unmet need and provides more evidence for the feasibility of external validation of a radiomics-based prognostic model. Our study addresses these pertinent research questions:

1. What performance increase does adding a set of radiomic features offer to a prognostic model, compared to only using clinical features?
2. Does a prognostic radiomics-based model offer equal predictive accuracy for BMs from different primary cancer types?
3. Do prognostic radiomics-based models predict outcomes of BMs with different volumes with varied accuracy?
4. Does the removal of volume-correlated features produce prognostic models with more balanced accuracy across BMs of different volumes?
5. Which clinical and radiomic features are most important for a prognostic model both before and after the removal of volume-correlated features?
6. What effect does the inclusion of multiple MR scanner models have on the accuracy of a radiomics-based model?

## Methods

### Study sample

We analyzed 99 patients from the cohort studied by Rodrigues et al.^7^ for whom MRI and BM contouring data were available. These 99 patients were randomly selected from the initial cohort to minimize selection bias. For the initial cohort, there were implicit inclusion and exclusion criteria reflective of clinical practice due to the retrospective design of the study by Rodrigues et al.^7^. Specifically, patients with highly symptomatic BMs or poor prognosis would be excluded from receiving SRS. For the retrospective study itself, the inclusion criteria consisted of patients with up to three newly diagnosed, radiologically confirmed BMs that were treated using SRS between 2003 and 2011. Patients with previous surgical resection, prior radiation therapy to the brain, or recurrent BMs were excluded. Due to the data requirements for MRI analysis and a radiologically confirmed endpoint, patients were also excluded if they did not have a pre-treatment or follow-up MRI.

The SRS treatments were performed using either the Novalis or Novalis TX linac with a Gill–Thomas–Cosman frame or BrainLAB frameless mask system for immobilization (BrainLAB, Feldkirchen, Germany). BMs < 7.5 cc generally received the most aggressive prescription (21 Gy in one fraction), while larger BMs would receive less aggressive prescriptions. This dose prescription guideline was developed by the centre at which the data were collected, as unified prescription protocols were not yet widely available. 127 BMs in total were treated with SRS. Four of these BMs were excluded as they consisted of a single voxel due to partial volume effects during application of the BM contouring data to the MRI voxel grid, and many radiomic features (e.g. all second-order texture features) would not be computable as they require more than one voxel. Outcome prediction and analysis was performed per BM instead of per patient, giving a total of n = 123 BMs that were individually analyzed. Table 1 shows the clinical features of the patients and BMs in this study.

**Table 1﻿:** Clinical feature distributions for number of BMs, BMs progressing post-SRS, and patients (where applicable) for the study sample.

Clinical features	# Patients	# BMs (% progression)
**Sex (*p* = 0.831)**		
Female	55	66 (21.2%)
Male	44	57 (22.8%)
**Age (*p* = 0.925)**		
Median (range)	58.0 (38.4–86.0) years	
**Primary cancer active (*p* = 0.002)**		
Yes	44	55 (9.1%)
No	55	68 (32.4%)
**Primary cancer site (*p* = 0.003)**		
Lung	59	70 (12.9%)
Breast	10	14 (35.7%)
Renal	10	15 (13.3%)
Colorectal	8	10 (40.0%)
Skin	8	9 (66.7%)
Other	4	5 (20.0%)
**Primary cancer histology (*p* = 0.001)**		
Adenocarcinoma	49	65 (20.0%)
NSCLC	31	36 (11.1%)
Melanoma	8	9 (66.7%)
Squamous carcinoma	7	8 (50.0%)
Other	4	5 (0.0%)
**Extracranial systemic metastases (*p* = 0.665)**		
Yes	39	50 (20.0%)
No	60	73 (23.3%)
**Systemic therapy status (*p* = 0.033)**		
Radical	51	10 (20.0%)
Palliative	41	60 (31.7%)
None	7	53 (11.3%)
**Neurological symptoms steroid response (*p* = 0.426)**		
Fully resolved	48	31 (16.1%)
Improvement	7	4 (50.0%)
Limited improvement	4	56 (25.0%)
No improvement	26	11 (9.1%)
Unknown	14	21 (23.8%)
**ECOG/WHO performance score (*p* = 0.580)**		
0	31	39 (17.9%)
1	60	73 (21.9%)
2	6	9 (33.3%)
3	2	2 (50.0%)
**GTV volume (*p* < 0.001)**		
Median (range)	3.07 (0.02–30.23) cc	
< 7.5 cc	–	94 (17.0%)
> 7.5 cc	–	29 (37.9%)
**BM location (*p* = 0.626)**		
Supratentorial	–	96 (22.9%)
Infratentorial	–	27 (18.5%)
**SRS prescription (*p* = 0.003)**		
15 Gy in 1 fraction	–	5 (0.0%)
18 Gy in 1 fraction	–	36 (30.6%)
21 Gy in 1 fraction	–	72 (13.9%)
24 Gy in 3 fractions	–	10 (60.0%)

The “Neurological Symptoms Steroid Response” feature qualitatively scores the improvement of neurological symptoms after the administration of steroids, based on the previously reported methodology of Lagerwaard et al.^24^. *p*-values provided for statistical comparisons between BMs that progressed and did not progress post-SRS. The Wilcoxon rank sum test was used for continuous features (age and GTV volume). The Chi-squared test was used for the remaining categorical features. *NSCLC* non-small cell lung cancer, *ECOG* Eastern Cooperative Oncology Group, *WHO* World Health Organization.

Each BM’s region-of-interest was defined as the gross tumour volume (GTV) manually contoured during SRS treatment planning by an experienced radiation oncologist, which was based on the outer border of the enhancing region in high-resolution T1w-CE MRI. Non-enhancing regions within the border were included in the contour. T1w-CE MRI was acquired with different MR scanner models, voxel resolutions, and acquisition orientations. Five scanner models from Siemens and General Electric (Erlangen, Germany; Chicago, USA) and eight scan configurations were used (Table 2).

**﻿Table 2:** Number of patients and BMs scanned by each of the MR scanner model and acquisition parameters configurations.

Scanner model and field strength	Acquisition orientation	Voxel size (mm^3^)	# Patients	# BMs (% progression)
Siemens Magnetom Vision (1.5 T)	Sagittal	1 × 1 × 1.5	35	39 (28.2%)
Siemens Avanto (1.5 T)	Sagittal	0.5 × 0.5 × 1	30	37 (13.5%)
	Axial	0.5 × 0.5 × 2	5	8 (0.0%)
Siemens Magnetom Expert (1.0 T)	Sagittal	1 × 1 × 1.5	21	29 (31.0%)
Siemens Sonata (1.5 T)	Sagittal	1 × 1 × 1.5	5	5 (40.0%)
	Axial	1 × 1 × 1.5	1	1 (0.0%)
	Axial	1 × 1 × 2	1	3 (0.0%)
General Electric Signa HDxt (1.5 T)	Sagittal	1 × 1 × 1.5	1	1 (0.0%)

Chi-squared test for progression yielded *p* = 0.226 across all scanner models and *p* = 0.069 across further investigated Vision, Avanto, and Expert scanners.

### Clinical features and study endpoint

For each patient, a set of 12 clinical features that were readily available for collection were used (Table 1), representing data that would be typically available in clinical practice before SRS.

The study endpoint was progression of each BM post-SRS, which was assessed on follow-up T1w-CE MRI acquired approximately every 3 months. Across the study sample, there was a median follow-up time of 9 months, with an interquartile range of 6 months, minimum of 4 months, and the two longest followed patients were last imaged at 16 and 24 months. BMs that had not progressed when lost to follow-up were scored as non-progression. For each BM, the maximum diameter was measured in three perpendicular directions (superior-inferior, mediolateral, posterior-anterior) by a single radiation oncologist. The product of the three maximum diameters was taken to provide a correlate to BM volume given the approximate spherical appearance of most BMs. If this quantity increased by ≥ 25% post-treatment, then the BM was recorded as having progressed. This endpoint was used to define a binary classification label for each BM, with BM progression defined as “positive”, and non-progression defined as “negative”. Our study contained 22.0% BMs that progressed, versus 78.0% that did not.

BMs can appear to progress in T1w-CE post-SRS but not be associated with cancerous progression. This is known as pseudo-progression^24^. We differentiated between true and pseudo-progression based on expert clinician judgement using post-SRS serial MRI and medical records. Pseudo-progression was scored as non-progression.

### Radiomic features

The T1w-CE MRI scans were pre-processed to account for voxel resolution and intensity scaling differences between MR scanner models. The mean and standard deviation of all voxels within the brain were computed to apply a Z-score normalization to the entire scan at three standard deviations^22,25^. The scan was then linearly interpolated to a voxel size of 0.5 × 0.5 × 0.5 mm^3^.

Within each BM, radiomic features were extracted from the pre-treatment T1w-CE MRI. 107 unique radiomic features were extracted using the open-source PyRadiomics library v3.0.1 in Python v3.6.13^26^ (list provided in Supplementary Table S1). Where required for feature extraction, 64 intensity value bins were used.

### Machine learning experiment design

Our ML experiments were performed using a study design template built in Matlab 2019b v9.7.0.1190202 (The Mathworks Inc., Natick, USA), consisting of a bootstrapped resampling design in which the dataset was partitioned into a training and testing dataset through random sampling with replacement. This was done at a per-patient level to avoid data leakage, by disallowing a patient’s BMs from being in the training and testing datasets.

The training dataset was used for inter-feature correlation filtering, hyper-parameter optimization, and training of a random decision forest model. This trained model was then evaluated on its predicted probabilities of BM progression in the testing dataset. After 250 iterations of bootstrapped resampling, we calculated the mean model predictive accuracy and associated confidence intervals. For reproducibility, a detailed explanation of the process is provided in Supplementary Fig. S1 and Table S2.

### Machine learning experiment error metric calculation

We computed a set of error metrics to describe the predictive accuracy of each technique. We used the predicted probabilities of progression and ground truth SRS outcomes, aggregated across all the bootstrapped repetitions’ testing datasets, to compute an average receiver operating characteristic (ROC) curve, area under the ROC curve (AUC), and associated 95% confidence intervals (CIs). It is well known that the average AUC from a bootstrapped resampling experiment is an underestimate of performance on unseen data^27^. To have AUC values that were more readily comparable to other studies, we also computed the commonly employed AUC_0.632+_ that was developed to correct for this underestimation^27^.

To calculate the average misclassification rate (MCR), false negative rate (FNR), and false positive rate (FPR), an operating point on the average ROC curve had to be chosen. The predicted probabilities from the out-of-bag datasets for each tree within each random decision forest were aggregated across all bootstrapped repetitions. From these aggregated probabilities, another average ROC curve was constructed and the optimal upper-left operating point found. The out-of-bag dataset probabilities were strictly informed by each repetition’s training dataset, and so provided an optimal operating point while avoiding overfitting to the testing dataset ROC curve. The optimal operating point was then transferred to the testing dataset ROC curve to compute the average MCR, FNR, and FPR. Supplementary Fig. S1 provides an illustration of this approach.

### Machine learning experiments addressing the research questions

To answer the first research question, on the relative predictive accuracy of clinical and radiomic features, three experiments were performed, each identical except for the features available to the model. The first experiment used only clinical features, the second used only radiomic features, and the third used all features.

The second research question, surrounding the effect of primary cancer type on predictive accuracy, was addressed through stratified calculation of error metrics by primary cancer type. The testing dataset and out-of-bag predicted progression probabilities from the previous experiment using both clinical and radiomic features were re-aggregated across bootstrap repetitions, with probabilities grouped according to the primary cancer site for each BM. These grouped probabilities were used to calculate the error metrics for each primary cancer site. This re-aggregation and grouping were required as some primary cancer types were associated with very few BMs, and so training models specifically for each primary cancer type was not feasible.

To investigate the effects of BM volume described in the third research question, similar techniques to that described above for stratified primary cancer type analysis were used, except based on BM volume. The BMs were stratified into one group of small BMs (< 7.5 cc), and a second group of large BMs (> 7.5 cc), to reflect the clinical use of the 7.5 cc volume threshold for dose prescription. Error metrics were then individually calculated for each BM volume group.

We answered the fourth research question on the effect of removing volume-correlated features by identifying features for removal using statistical tests for dependence on and correlation to BM volume. For categorical clinical features, the dependence of a feature on BM volume was determined using the Wilcoxon rank sum test or Kruskal–Wallis test, depending on whether the feature was binary or not. If a feature was found to have a *p*-value less than a Bonferroni-corrected alpha value of α = 0.05/118, it was removed. Pearson correlation coefficient values were computed between continuous features and both BM volume and its cubic root (analogous to BM diameter). If the *p*-values associated with the correlation coefficients of a feature was significant at α = 0.05/118, then the feature was available for removal if either correlation coefficient was greater than a given correlation coefficient threshold. Correlation coefficient thresholds between 0.1 and 0.85 at a spacing of 0.15 were tested, along with a threshold of 0. This methodology was designed to explore gradually blinding the model to BM volume to result in more balanced predictive accuracy between the two volume groups.

The fifth research question addressing the relative importance of clinical and radiomic features in the prognostic models was answered using feature importance rankings inherent to random decision forest models. To aggregate the feature importance scores across bootstrapped repetitions, the raw feature importance scores for each repetition were normalized to be between 0 and 1, and any features removed by the inter-feature correlation filter (as opposed to the volume correlation filter) were given a feature importance score of 0. The scores were then averaged across all bootstrapped repetitions and then renormalized to be between 0 and 1. A feature was deemed “highly important” if it had a final feature importance score above 0.75.

Lastly, to address the final research question around the effects of MR scanner variability, repeat experiments were performed with datasets stratified by MR scanner model. For the study sample, most patients were imaged using either the Magnetom Vision, Magnetom Expert, or Avanto scanner models (see Table 2). Three repeat experiments using all radiomic and clinical features were then performed with a subset of the study sample that was imaged only on two of these three scanner models. A relatively high predictive accuracy from one of these experiments would suggest the two scanners being in a similar data domain, while a low predictive accuracy would suggest the opposite.

### Ethics declaration

The collection and analysis of the retrospective patient data used in this study was approved by the Amsterdam University Medical Centre Medical Ethical Review Committee and was conducted within the approved guidelines. As the study was retrospective on a cohort of deceased patients, written consent from study participants was waived by the Amsterdam University Medical Centre Medical Ethical Review Committee.

## Results

### Clinical and radiomic features predictive accuracy

**Figure 1:**
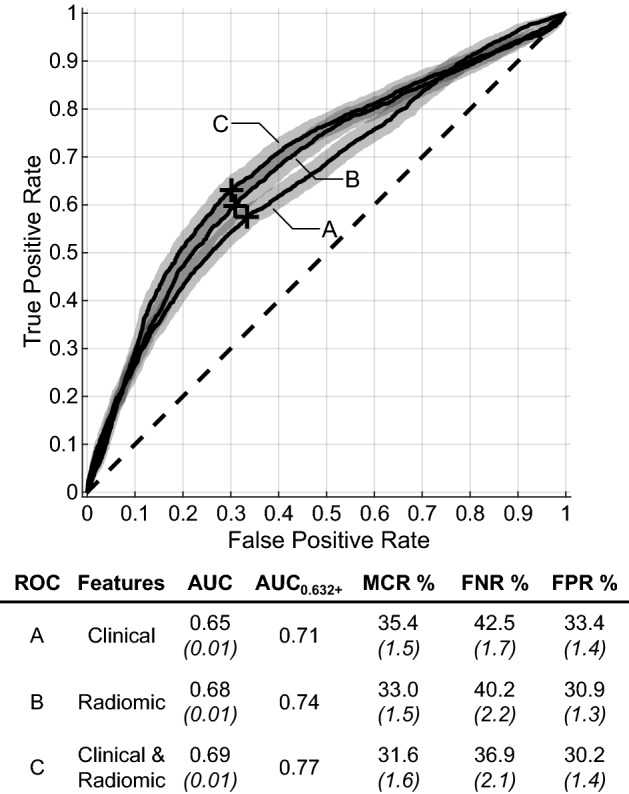
Comparison of average ROC curves across bootstrapped repetitions when clinical, radiomic, or clinical and radiomic features were used. The AUC for each curve is provided, along with the corrected AUC_0.632+_ value. The optimal upper-left operating point determined from the out-of-bag dataset is plotted as “+”, for which the MCR, FNR, and FPR were found. A false negative represents a BM that was predicted to respond to SRS, but instead progressed after SRS. 95% confidence intervals are provided for the curves (shaded bands) and error metrics (in parentheses).

Figure 1 shows the average ROC curves comparing the performance of using clinical features alone, radiomic features alone, and their combination. Comparison of the AUCs reveals that clinical and radiomic features offer the superior prognostic model. While the AUC differences between the scenarios are statistically significant, they are small (0.01 to 0.03). Given these results, we used clinical and radiomic features for the remainder of the experiments.

The reported AUC values are likely pessimistically biased values due to the nature of the bootstrapped resampling technique. In Fig. 1, the AUC_0.632+_ consistently shows a lift in AUC, up to AUC = 0.77 when clinical and radiomic features are used. The small 95% CIs demonstrate the consistent performance of our technique across repetitions.

Figure 1 also contains the MCR, FNR, and FPR values for each ROC curve. They show that FPR remains stable between feature types, with higher AUC values correlating with lower FNRs.

### Primary cancer site dependency of predictive accuracy

The predictive accuracy of the model varied widely between primary sites when using clinical and radiomic features (Table 3). Progression is predicted most accurately for colorectal and skin, while renal and lung were predicted with AUCs closer to the overall accuracy across all sites. For breast cancer primaries, predictive accuracy was at chance.

**﻿Table 3:** Error metrics derived by analysis per BM primary cancer site.

Primary site	AUC	MCR %	FNR %	FPR %
Colorectal	0.90 *(0.04)*	19.8 *(3.6)*	15.8 *(3.3)*	20.9 *(3.7)*
Skin	0.84 *(0.05)*	27.4 *(4.5)*	17.8 *(3.2)*	30.1 *(4.9)*
Renal	0.72 *(0.03)*	39.1 *(3.2)*	29.1 *(4.9)*	41.9 *(2.8)*
Lung	0.64 *(0.02)*	35.5 *(2.0)*	45.2 *(3.3)*	32.7 *(1.7)*
Breast	0.49 *(0.04)*	41.5 *(3.1)*	48.9 *(4.0)*	39.4 *(2.9)*

95% confidence intervals provided in parentheses.

The 95% CIs of the error metrics were found to vary across primary sites. Lung cancer was the most common primary site, yielding more data points to determine the average ROC curve, reducing the associated CIs.

### Volume dependency of predictive accuracy

**Figure 2:**
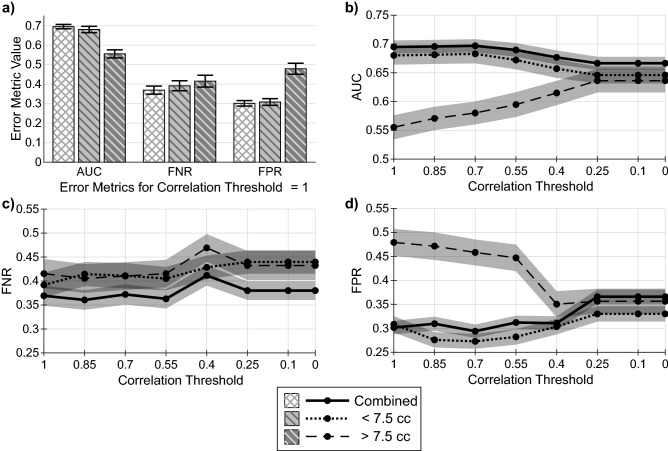
Volume dependency of error metrics. (**a**) Shows the comparison of error metrics between all BMs, BMs with volume < 7.5 cc, and BMs with volume > 7.5 cc before the removal of any volume-correlated features. (**b**–**d**) show the AUC, FNR, and FPR for each BM volume group as a function of the feature volume-correlation coefficient threshold value used to remove continuous features, with correlation threshold = 1 corresponding to the removal of no features (baseline). At all correlation thresholds < 1, categorical clinical features dependent on BM volume are also removed. For example, values at correlation threshold = 0.7 represent the error metric values when the experiment was repeated with all features with volume-correlation coefficient values greater than 0.7 removed. For all values, a 95% confidence interval is provided by error bars or shaded bands.

Our technique had varying predictive accuracy depending on BM volume when using clinical and radiomic features. For small BMs (< 7.5 cc) we found that response to SRS was predicted with accuracy comparable to using the overall dataset (Fig. 2a). Performance for large BMs (> 7.5 cc) fell considerably with statistical significance. The lower AUC value for large BMs mostly translated into a higher FPR, nearing 50%. Large BMs had the widest 95% CIs, likely due to their relatively small number.

The only clinical feature found to be dependent on BM volume, and therefore removed, was the received dose and fractionation. In total, 72 features were removed at the correlation threshold of 0.25, leaving 47 features for use by the model. As the threshold was reduced past 0.25, no further features were candidates for removal due to their level of statistical significance. As volume-correlated features were removed down to a correlation threshold of 0.25, we found the difference in AUC, FNR and FPR for small and large BMs was reduced to within the associated 95% CIs. Figure 2b–d demonstrates that while FNR remains well-matched across volume groups, FPR fell and AUC rose significantly for large BMs, while the metrics for small BMs and the overall dataset remained stable. As no further features were removed past a threshold of 0.25, model performance remained constant past the 0.25 threshold as well.

### Feature importance analysis

The relative importance of all the clinical and radiomic features at each correlation coefficient threshold are provided within Supplementary Fig. S2. Supplementary Table S3 provides a detailed comparison between the highly important features at the correlation threshold of 1 and 0.25, but the key results are provided here.

Primary cancer site and histology were highly important before and after volume-correlated features were removed. Before volume-correlated features were removed, primary cancer site and histology were the second and fourth most important features, respectively, and were the third and fourth most important features when the correlation threshold was 0.25. The only other highly important clinical feature was GTV volume, but only before it was removed. Univariate analysis showed that the “primary cancer active” and “systemic therapy status” features predicting post-SRS progression with p < 0.05 (Table 1). These two features were not found to be important by the model however, suggesting that alternative features provided superior predictive value.

When the correlation threshold was set to 0.25, eight radiomic features were highly important, and they gained importance as more volume-correlated features were removed. Four of the eight radiomic features, gray-level co-occurrence matrix (GLCM) cluster shade, GLCM cluster prominence, GLCM contrast, and interquartile range, were found to transition to become highly important features as the correlation threshold was reduced to 0.25. The remaining four features (kurtosis, GLCM inverse variance, neighbouring gray tone difference matrix contrast, and 10^th^ percentile value) consistently remained highly important as features were removed. As volume-correlated features were removed to reach the 0.25 threshold, 11 radiomic features that were highly important before feature removal were found to be correlated to BM volume or diameter.

### Effects of multiple magnetic resonance scanner models

**Figure 3:**
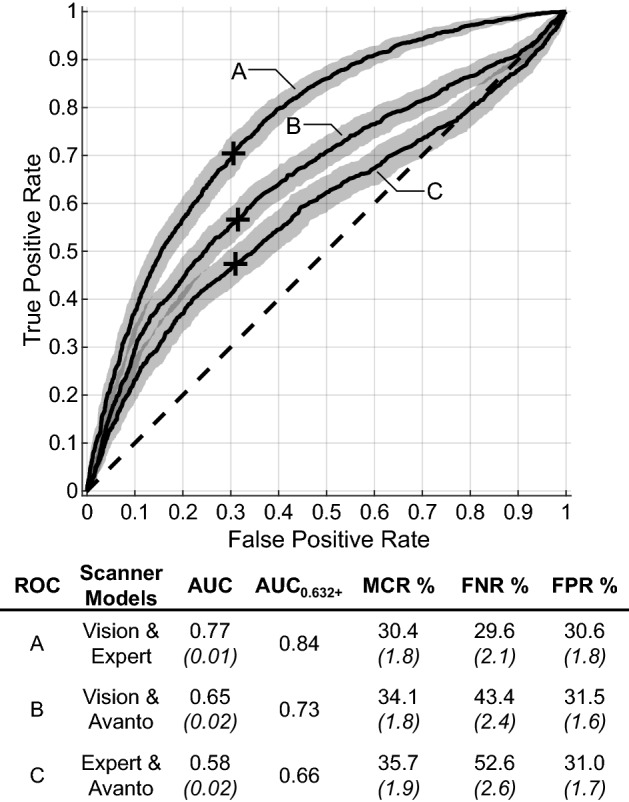
Error metrics across MR scanner models. The average ROC curves across bootstrapped repetitions are compared when using clinical and radiomic features only from pairings of the three primary MR scanner models used (Siemens Magnetom Vision, Magnetom Expert, and Avanto). As for Fig. 1, for each curve in this figure, associated error metrics, optimal operating point (+), and 95% confidence intervals (shaded band and values in parentheses) are provided.

The pairing of the Magnetom Vision and Expert scanners yielded increased predictive accuracy; AUC increased (from a baseline of 0.69), translating mostly into a decrease of FNR (Fig. 3). When the Avanto scanner was paired with either the Magnetom Vision or Expert scanner, the performance of the model was found to decline when compared to the baseline.

## Discussion

Our study showed the synergistic relationship of clinical and radiomic features. Previous studies have reported on the relative accuracy of clinical and radiomic features in BM SRS; however, the disagreement of their results may be due to varying experimental designs. Mouraviev et al. observed an 18% AUC increase between using clinical features alone to using radiomic and clinical features, though using radiomic features alone was not investigated^12^. Jiang et al. saw no increase in AUC when using radiomic features alone compared to using radiomic and clinical features, though no baseline of using clinical features alone was established^15^. Studies on post-operative BM SRS or SRT found radiomic features outperformed clinical features and that clinical features decreased accuracy when combined with radiomic features^17,18^.

Our study presents a more robust comparison of clinical and radiomic features than previously reported. All previous studies used fewer clinical features than investigated here. Some studies selected radiomic features using the entire dataset, leading to possible overfitting^12,19^. All studies selected radiomic features in the absence of the clinical features, leading to possibly rejecting radiomic features that are only beneficial in conjugation with clinical features. By including more clinical features and performing repeated experiments including clinical and/or radiomic features, our study provides a firmer result that the use of clinical and radiomic features together does indeed offer increased accuracy, but not as large of a benefit as previously reported.

Different primary cancer sites have been associated with varying BM SRS success rates^2,3,28^. Our study offers a subtly alternative and unexplored insight that the outcomes of BMs from some primary sites are more difficult to predict than others with a model trained with data from multiple primary sites. Due to small sample sizes of some primary sites and training the model on all primary sites, it is difficult to make strong conclusions as to why this occurs. Radiomic features of BMs have been shown to be moderately predictive of primary cancer site, with AUCs up to 0.87^29^. There could therefore be radiomic features unique to certain primary cancer types that are useful for outcome prediction. Our small primary cancer subsamples and the fact that no other studies have provided similar analysis as presented here make probing this hypothesis difficult. Future study of this question with sufficient sample size is warranted. We also explored controlling for primary cancer site in a similar manner as was used for controlling for the BM volume effect by removing any features found to be dependent on primary cancer site as determined through Kruskal–Wallis and Chi-Squared tests and a Bonferroni-corrected α = 0.05/118. The primary cancer active, primary cancer histology, and systemic metastases clinical features were all dependent on primary cancer site and were removed along with primary cancer site. The same experiments and analysis were then conducted and a negligible impact on the error metrics per primary cancer site was found, leaving the disparity between primary cancer sites intact.

Our results indicate that externally validating a model on a sample with differing primary cancer ratios than the model’s training sample could lead to difficulty interpreting the results. For example, our technique would perform quite poorly if validated with a sample containing a high incidence of breast cancer. Furthermore, such imbalanced performance could lead models to provide little benefit to patients with certain primary cancers, despite promising overall performance. Performance evaluation per primary cancer site should therefore become standard practice.

This performance imbalance could be addressed by constructing distinct models for each primary site. Radiomic models have been developed for SRS treating BMs from lung^15^ and skin^14^ primary cancers, and they yield higher accuracy metrics than every radiomic study on mixed primary cancers. Alternatively, our techniques appear to offer a very useful model for colorectal or skin cancer patients as is, and so training a model using data with mixed primary cancers may still offer high value to some patient sub-populations. Performing a similar evaluation based on the primary cancer histology and developing histology specific models could also be a critical avenue for future exploration. As primary cancer histology is typically known for a BM prior to SRS treatment, histology specific models would likely be as clinically translatable as models that are primary site specific.

SRS is well known to be less likely to fail for smaller BMs^2^. This is reflected in our study sample with GTV volume being the only clinical variable providing a univariate prediction of SRS outcome with p < 0.001. Therefore, a model that predicts the response of small BMs well, but not large BMs, lacks clinical utility.

The correlation of many radiomic features with region-of-interest size^20,21^ (BM volume or diameter in our study) also demonstrates a challenge for predicting BM SRS outcomes. When comparing the 74% of radiomic features that were volume-correlated in this study to the important features identified in other BM SRS studies, it was found that 60–73% of important features reported elsewhere were likely volume-correlated^12–14,16^. The exception was the study of Jiang et al., but since their methodology rejected BMs < 10 mm in diameter and did not find volume to be a univariate predictor of SRS response, it is not surprising that no volume-correlated radiomic features were found to be important^15^. Gutsche et al. removed volume-correlated radiomic features, but only at a correlation coefficient > 0.9, and so retained many features that remained strongly correlated with volume^16^.

Our study found that by effectively blinding the model to BM volume through the removal of volume-correlated features, balanced accuracy for small and large BMs could be achieved. This result may initially appear counterintuitive. One would wonder: if BM volume is such a strong univariate predictor of SRS outcomes, why does removing it and other volume-correlated features not significantly decrease accuracy? The reason for more balanced performance between small and large BMs is similarly unclear. We speculate that the reason for this is that small BMs receive more aggressive prescriptions and are less hypoxic/sufficiently vascularized only in general. Therefore if the model is blinded to volume, but still has access to direct information about hypoxia/vascularization through the remaining radiomic features, overall accuracy should not fall. Furthermore, by only having these features more specific linked to SRS response, we speculate that the model would tend towards making more nuanced predictions for large BMs.

We identified an optimal correlation threshold of 0.25; with a less strict threshold (> 0.40), accuracy remained imbalanced between the volume groups, and with a stricter threshold (< 0.1), no further features were identified for removal. The volume-correlation threshold of 0.25 needs to be externally validated in future work. It should also be noted that our results were presented only for a single, but clinically relevant, volume threshold of 7.5 cc. While this volume threshold was relevant at the centre at which the study data were collected, external validation of this threshold, or thresholds more relevant at an external centre, is also required.

In our study, novel and previously reported radiomic features were found to predict BM SRS outcomes. Of the eight highly important radiomic features not correlated to volume, two were also found to be highly important in other studies: GLCM cluster shade^14^ and kurtosis^13,16^. This is the first report of multiple radiomics features being reproducibly identified as important across more than one centre for predicting BM SRS response. The remaining six highly important radiomic features were newly identified; their importance may be unique due to our sample, removal of volume-correlated features, or MRI pre-processing.

Interpretation of radiomic features, and especially their complex interactions within a ML model, is notoriously difficult. Previous literature has linked qualitative BM appearance and SRS outcomes^7,8^. While this study has linked some radiomic features with BM progression post-SRS, future study should more directly link radiomic features with qualitative BM appearance to aid in interpretability.

The accuracy variability observed across scanner models shows that not all scanner differences could be normalized by our methodology. We speculate that the different native resolution of the Avanto scanner compared to the Expert and Vision scanners explains the decrease in accuracy when using Avanto scanner images. A dataset with more scanner models and resolutions would be required to confirm this. The majority of the Avanto scanner images (37 BMs) were acquired sagittally, but 8 BMs were acquired as slightly thicker axial slices (Table 2). The Expert and Vision scanners exclusively acquired sagittal slices. To ensure this difference was not a factor in the observed accuracy variability, the experiments were repeated omitting the 8 BMs acquired axially. These results were consistent with those presented previously, indicating that mixed slice orientations did not cause the accuracy variability.

Our findings on the effects of mixed MR scanner models are relevant to clinical translation. When using only the more closely matched Expert and Vision scanners, we achieved AUC 0.84 despite using half of our study sample (68 BMs). This AUC exceeds or is comparable to the current state of the field. Given the drop in accuracy when including the Avanto scanner, it is likely that current literature results are optimistic compared to expected results during external validation using a different scanner model. Some studies in this area have used datasets with more than one scanner^13,16,17^. Mulford et al. employed a 1.5 T and 3 T scanner, but 96% of the data was from the 1.5 T scanner, likely masking any 3 T scanner effects. Studies by Wang et al. and Gutsche et al. also used a 1.5 T and 3 T scanner, but in both cases the ratio of data from each scanner and scanner model analysis were not provided, making drawing conclusions challenging. The magnitude of the drop in accuracy when including Avanto scanner is also important to consider. AUC was found to decrease up to 13.0% when introducing the Avanto scanner, which exceeds the AUC changes observed when altering the available features. It is therefore critical to characterize and correct for the effect of MR scanner variability and not only the available features, as has been the standard practice to date in the literature.

Our study’s methodology was unable to normalize for differences across all scanner models, but further techniques could be investigated. The instability of radiomic features across MR scanner models has been previously reported^22,23,30,31^. Research into producing more generalizable models across imaging scanners has shown the success of pre-processing normalization, post-feature extraction harmonization, and data augmentation^22,30–32^. The Z-score transform performed in this study is but one of these techniques, though was not found to operate optimally. A robust comparative study of these techniques is required to produce a model that will more likely maintain performance across multiple centres or can be calibrated for use per MR scanner.

It is important to also consider the limitations of this study with respect to the clinical endpoint considered. First, this study examined only the endpoint of per BM post-SRS progression, whereas prediction of progression free survival and overall survival may also hold clinical utility. While BM MRI radiomics are the most likely to be linked to per BM progression, radiomics of the entire brain, primary cancer, and extra-cranial metastases may enhance predictions of these additional per patient endpoints. Second, to definitively differentiate pseudo-progression against true progression post-SRS, biopsy would need to be performed on each BM that progressed, as reliable imaging techniques to distinguish pseudo-progression from true progression are not yet available^33^. This study did not use this approach due to its retrospective nature, as biopsies to confirm pseudo-progression are not routinely gathered for all patients in standard clinical practice. While prospective studies of SRS outcome modeling would benefit from more robust pseudo-progression diagnostic techniques as outlined in the Response Assessment in Neuro-Oncology Brain Metastases (RANO-BM) protocol^33^, we believe our study’s methodology still provides insight into SRS outcome prediction that could motivate these prospective studies. Third, this study’s endpoint measurements were performed before the publication of the RANO-BM protocol^33^. The volumetric measurement then used in this study does provide a more comprehensive description of the change in BM size compared to the RANO-BM protocol, but its inability to be directly compared to studies utilizing RANO-BM does provide a limitation to this study. Fourth, another limitation of this study’s endpoint is the variable follow-up time for each patient due the short overall survival for many BM patients. This shortcoming is inherent to all BM studies, as by definition all BM patients are in the most advanced stage of their cancer. Patients lost to follow-up early on may then have had BMs that would have progressed if the patient had survived long enough, but because of discontinued follow-up, the BMs would be scored as non-progression. This limitation therefore affects the dataset’s endpoints for some metastases, and so the results presented here, and in the field more broadly, should be viewed as conservative compared to expected results if the true endpoint was known for each BM.

This study was also limited due to constraints of its retrospective design. First, as with many radiomics ML studies, the small, single centre, retrospective study sample limit the strength of the conclusions. Second, the study sample’s patients were treated using a linac between 2003 and 2011. While this study presents the first results on SRS outcome prediction performed on a general purpose linac (whereas previous studies examined only Gamma Knife or Cyber Knife), the results may not generalize to alterative or more recent SRS treatment modalities. Third, this study only utilized clinical features that were readily available at the institution where the data was collected, and so the results presented are specific to these clinical features and associated scorings. While this study offered the widest variety of clinical features to date in the field, there are further clinical features to examine that may hold promise. In particular, biomarkers specific to certain primary cancer sites could present unique opportunities if used with site specific models. Fourth, while the bootstrapped experimental design used is a robust technique free of data leakage between the training and testing datasets, the use of an independent testing dataset would ensure that overfitting to the dataset was prevented. We are currently conducting a study at a separate centre that will address these limitations. This study will contain a larger and more recent sample of linac-based SRS patients. This study will allow for external validation of the models produced here and further investigation of MR scanner model variation.

In conclusion, this study showed that predictive accuracy is highest when using clinical and radiomic features together, and depends highly on primary cancer site, BM volume and the inclusion of multiple MR scanner models. We found that performance was particularly high for colorectal and skin cancer patients, and so our techniques may be of more benefit to these sub-populations. We also showed that accuracy dependency on BM volume can be eliminated without sacrificing overall accuracy by removing volume-correlated features, with radiomic features uncorrelated with volume becoming more important. We believe before such prognostic models can be externally validated or clinical translated, it is imperative that these effects be analyzed, understood, and accounted for in future exploratory studies. By doing so, we believe that prognostic models suitable for clinical implementation can be produced that can aid clinicians in prescribing stereotactic radiosurgery to minimize risk to patients and brain metastasis progression post-treatment.

## Supplementary Information

Supplementary Information.

## Data Availability

The ground truth labels, sample stratifying variables, and predicted progression probabilities from each reported experiment’s models to replicate this study’s analysis are available for use at the following URL: https://github.com/baines-imaging-research-laboratory/radiomics-for-srs-performance-sensitivity-data-share.

## Code availability

All the computer code used for the study’s experiments, analysis and figure generation are available online at: https://github.com/baines-imaging-research-laboratory/radiomics-for-srs-performance-sensitivity-code.
