# Supplementary material for: Performance sensitivity analysis of brain metastasis stereotactic radiosurgery outcome prediction using MRI radiomics
Source: Sci Rep. 2022 Dec 5;12:20975. doi: 10.1038/s41598-022-25389-7 (PMC9722896; doi:10.1038/s41598-022-25389-7)
Supplement: Supplementary file 1 — Supplementary Information. [file 41598_2022_25389_MOESM1_ESM.pdf]

Performance Sensitivity Analysis of Brain Metastasis Stereotactic Radiosurgery Outcome Prediction using MRI Radiomics

Supplementary Material

David A. DeVries[1,2,*], Frank Lagerwaard[3], Jaap Zindler[4,5], Timothy Pok Chi Yeung[6], George Rodrigues[7], George Hajdok[1], Aaron D. Ward[1,2]

[1]Western University, Department of Medical Biophysics, London, N6A 3K7, Canada
[2]Gerald C. Baines Centre, London Health Sciences Centre, London, N6A 5W9, Canada
[3]Amsterdam University Medical Centre, Department of Radiation Oncology, Amsterdam, 1081, The Netherlands
[4]Haaglanden Medical Centre, Department of Radiation Oncology, Den Hague, 2512VA, The Netherlands
[5]Holland Proton Centre, Delft, 2629JA, The Netherlands
[6]RefleXion Medical, Hayward, 94545, USA
[7]Western University, Department of Oncology, London, N6A 3K7, Canada
*ddevrie8@uwo.ca

Table S1. Complete catalogue of the 119 features included within the study. The number for each feature is the "Feature Look-up Number" used elsewhere such as Figure S2 and Table S3. All features not with feature type "Clinical" are radiomic features computed from the pre-treatment T1w-CE MRI, with complete documentation of the features provided by the PyRadiomics project (https://pyradiomics.readthedocs.io/en/latest/features.html). Underlined numbers are only to aid the reader in looking-up feature numbers. Abbreviations: Gray-Level Co-occurrence Matrix (GLCM), Gray-Level Run Length Matrix (GLRLM), Gray-Level Dependence Matrix (GLDM), Gray-Level Size Zone Matrix (GLSZM), Neighbouring Gray Tone Difference Matrix (NGTDM).

#	Feature Type	Feature Name
1	First Order	10th Percentile
2	First Order	90th Percentile
3	First Order	Energy
4	First Order	Entropy
5	First Order	Interquartile Range
6	First Order	Kurtosis
7	First Order	Maximum
8	First Order	Mean Absolute Deviation
9	First Order	Mean
**10**	First Order	Median
11	First Order	Minimum
12	First Order	Range
13	First Order	Robust Mean Absolute Deviation
14	First Order	Root Mean Squared
15	First Order	Skewness
16	First Order	Total Energy
17	First Order	Uniformity
18	First Order	Variance
19	Shape & Size	Elongation
**20**	Shape & Size	Flatness
21	Shape & Size	Least Axis Length
22	Shape & Size	Major Axis Length
23	Shape & Size	Maximum 2D Diameter Column
24	Shape & Size	Maximum 2D Diameter Row
25	Shape & Size	Maximum 2D Diameter Slice
26	Shape & Size	Maximum 3D Diameter
27	Shape & Size	Mesh Volume
28	Shape & Size	Minor Axis Length
29	Shape & Size	Sphericity
**30**	Shape & Size	Surface Area
31	Shape & Size	Surface Volume Ratio
32	Shape & Size	Voxel Volume
33	GLCM	Autocorrelation
34	GLCM	Cluster Prominence
35	GLCM	Cluster Shade
36	GLCM	Cluster Tendency
37	GLCM	Contrast
38	GLCM	Correlation
39	GLCM	Difference Average
**40**	GLCM	Difference Entropy
41	GLCM	Difference Variance
42	GLCM	Inverse Difference
43	GLCM	Inverse Difference Moment
44	GLCM	Inverse Difference Moment Normalized
45	GLCM	Inverse Difference Normalized
46	GLCM	Informational Measure of Correlation 1
47	GLCM	Informational Measure of Correlation 2
48	GLCM	Inverse Variance
49	GLCM	Joint Average
**50**	GLCM	Joint Energy
51	GLCM	Joint Entropy
52	GLCM	Maximal Correlation Coefficient
53	GLCM	Maximum Probability
54	GLCM	Sum Average
55	GLCM	Sum Entropy
56	GLCM	Sum Squares
57	GLRLM	Gray-Level Non-Uniformity
58	GLRLM	Gray-Level Non-Uniformity Normalized
59	GLRLM	Gray-Level Variance
**60**	GLRLM	High Gray-Level Run Emphasis
61	GLRLM	Long Run Emphasis
62	GLRLM	Long Run High Gray-Level Emphasis
63	GLRLM	Long Run Low Gray-Level Emphasis
64	GLRLM	Low Gray-Level Run Emphasis
65	GLRLM	Run Entropy
66	GLRLM	Run Length Non-Uniformity
67	GLRLM	Run Length Non-Uniformity Normalized
68	GLRLM	Run Percentage
69	GLRLM	Run Variance
**70**	GLRLM	Short Run Emphasis
71	GLRLM	Short Run High Gray-Level Emphasis
72	GLRLM	Short Run Low Gray-Level Emphasis
73	GLDM	Dependence Entropy
74	GLDM	Dependence Non-Uniformity
75	GLDM	Dependence Non-Uniformity Normalized
76	GLDM	Dependence Variance
77	GLDM	Gray-Level Non-Uniformity
78	GLDM	Gray-Level Variance
79	GLDM	High Gray-Level Emphasis
**80**	GLDM	Large Dependence Emphasis
81	GLDM	Large Dependence High Gray-Level Emphasis
82	GLDM	Large Dependence Low Gray-Level Emphasis
83	GLDM	Low Gray-Level Emphasis
84	GLDM	Small Dependence Emphasis
85	GLDM	Small Dependence High Gray-Level Emphasis
86	GLDM	Small Dependence Low Gray-Level Emphasis
87	GLSZM	Gray-Level Non-Uniformity
88	GLSZM	Gray-Level Non-Uniformity Normalized
89	GLSZM	Gray-Level Variance
**90**	GLSZM	High Gray-Level Zone Emphasis
91	GLSZM	Large Area Emphasis
92	GLSZM	Large Area High Gray-Level Emphasis
93	GLSZM	Large Area Low Gray-Level Emphasis
94	GLSZM	Low Gray-Level Zone Emphasis
95	GLSZM	Size Zone Non-Uniformity
96	GLSZM	Size Zone Non-Uniformity Normalized
97	GLSZM	Small Area Emphasis
98	GLSZM	Small Area High Gray-Level Emphasis
99	GLSZM	Small Area Low Gray-Level Emphasis
**100**	GLSZM	Zone Entropy
101	GLSZM	Zone Percentage
102	GLSZM	Zone Variance
103	NGTDM	Busyness
104	NGTDM	Coarseness
105	NGTDM	Complexity
106	NGTDM	Contrast
107	NGTDM	Strength
108	Clinical	Gender
109	Clinical	Age
**110**	Clinical	Primary Cancer Active
111	Clinical	Primary Cancer Site
112	Clinical	Primary Cancer Histology
113	Clinical	Systemic Metastases Status
114	Clinical	Systemic Therapy Status
115	Clinical	Steroid Status
116	Clinical	WHO Score
117	Clinical	Gross Tumour Volume (GTV) Volume
118	Clinical	Brain Metastasis (BM) Location
119	Clinical	Dose and Fractionation

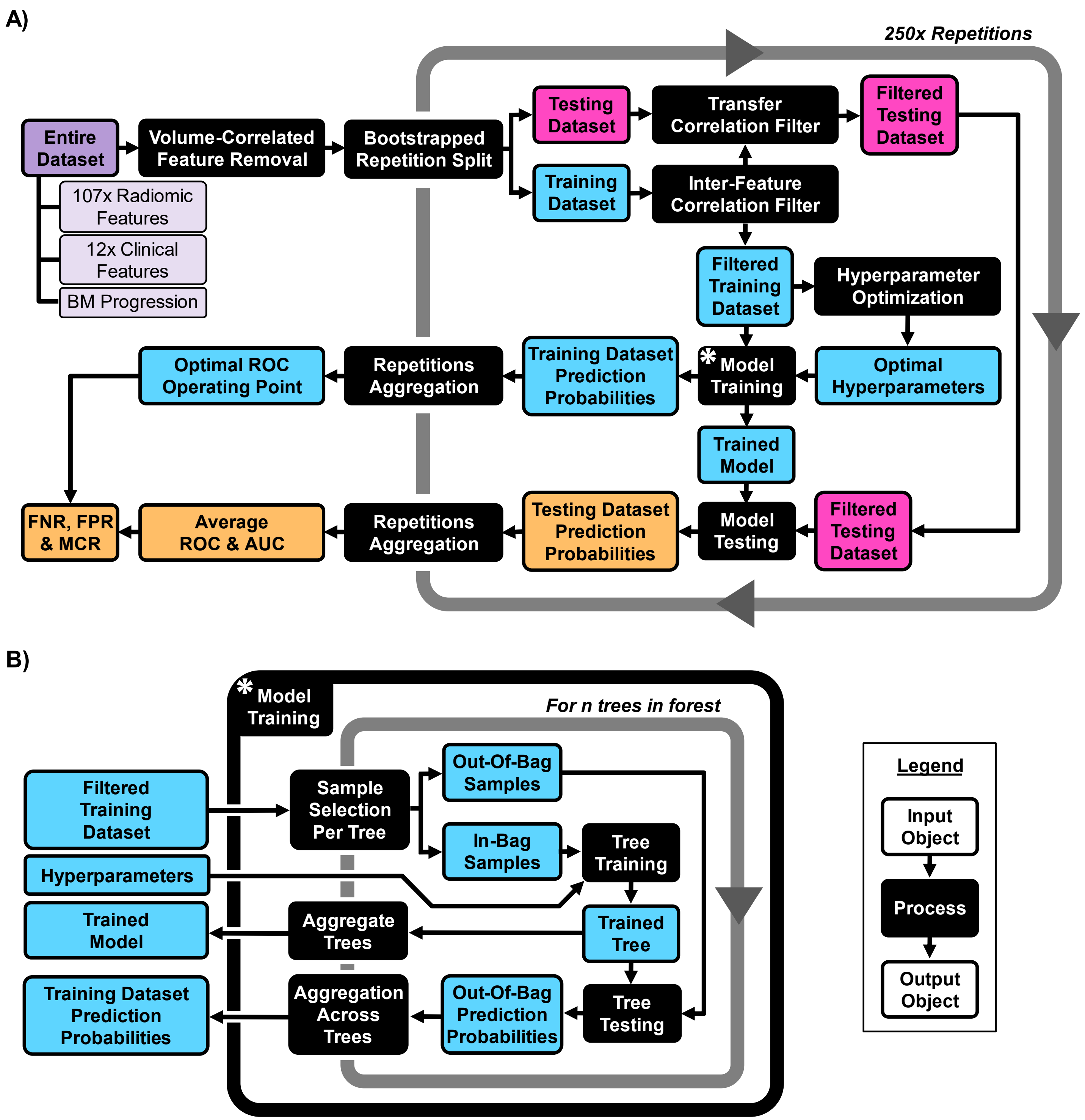

Figure S1. Schematic diagram of the machine learning experimental method technique used in the study. A) shows the overall experimental method, while B) provides enhanced detail of the model training to show how the out-of-bag samples from each tree in the random decision forest are used to produce the aggregated training dataset prediction probabilities. The colouring of the objects in the diagram is used to illustrate the separation of the entire dataset into training and testing datasets, the isolation of objects derived from each of the datasets during feature filtering and hyperparameter optimization (to prevent overfitting), and the recombination of objects from the datasets only during model testing and error metrics calculation. The inter-feature correlation filter used hierarchical clustering on the training dataset alone to determine groups of correlated features with a correlation coefficient >0.8. For each group of correlated features, the feature most strongly correlated to BM progression was retained. This filtering of features was then transferred to the testing dataset, ensuring the testing dataset did not inform the selected features. For the experiments investigating primary cancer site and BM volume effects, the methodology shown would remain identical, except that after the “Repetitions Aggregation” processes shown in A), the training and testing dataset prediction probabilities would be grouped by the associated BMs’ primary cancers and volumes. This would lead to an optimal ROC operating point, and average ROC, AUC, MCR, FNR, and FPR per primary cancer or volume group.

Table S2. Hyperparameters for the random decision forest model used. For hyperparameters that underwent optimization, the optimization domain and search transform are provided. Numerical domains are indicated with minimum and maximum values in square brackets. Hyperparameter optimization was performed using 50 iterations of Bayesian optimization using the expected-improvement-plus acquisition function. The AUC on the out-of-bag samples was used as the optimization objective function. For hyperparameters that were not optimized, their value and justification are provided. For further descriptions of the hyperparameters, see the documentation for the *TreeBagger* function provided in Matlab 2019b.

**Hyperparameter**	**Optimization Domain**	**Optimization Domain Search Transform**
Number of trees	[10, 1000]	logarithmic
Number of features to sample	[1, number of features]	linear
Minimum leaf size	[1, number of features / 2]	logarithmic
Maximum number of decision splits	[1, number of samples – 1]	logarithmic
Feature selection	curvature, interaction curvature	categorical
Decision split criterion	Gini’s diversity index, deviance	categorical
	**Value**	**Justification**
In-bag fraction	1	produces in-bag dataset that is the same size as the training dataset
Sample with replacement	on	allows for out-of-bag samples to be reserved for evaluating trained model using the training dataset
Cost per SRS response	equal for each response	false negatives and positives given equal cost
Prior	empirical	allows priors to be optimized for the study population
Algorithm for categorical features	exact	all combinations of categories for categorical features investigated at decision splits
Merge leaves	off	leaf merging not needed as trees are not pruned
Prune	off	tree pruning not needed as the maximum number of decision splits hyperparameter was optimized
Surrogate decision splits	10	not all surrogate splits investigated to decrease model training time
Weights	equal for each sample	all samples given equal importance during training

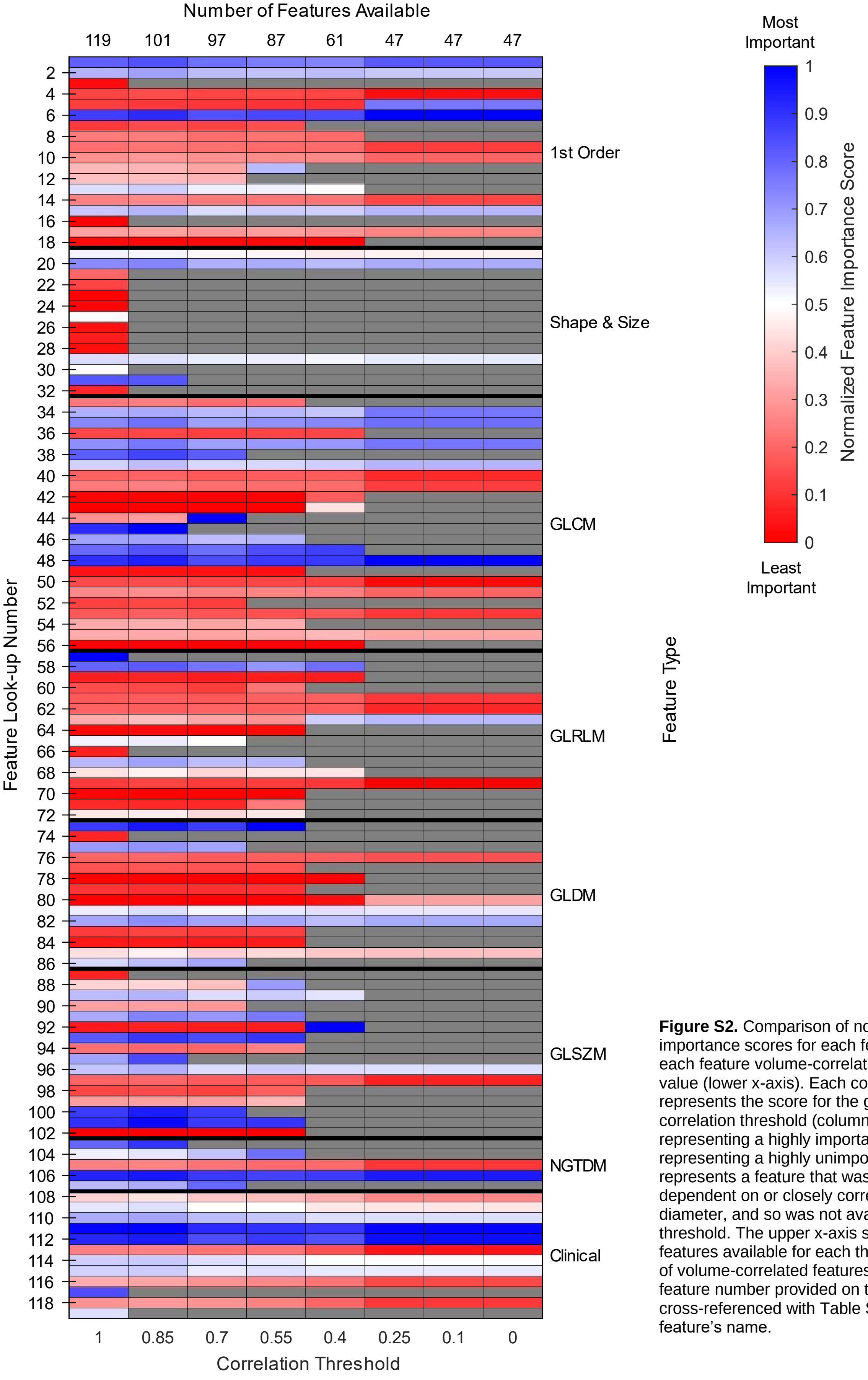

Figure S2. Comparison of normalized feature importance scores for each feature (y-axis) and for each feature volume-correlation coefficient threshold value (lower x-axis). Each colour-coded box represents the score for the given feature (row) and correlation threshold (column), with blue (1) representing a highly important feature and red (0) representing a highly unimportant feature. A grey box represents a feature that was removed due to it being dependent on or closely correlated to BM volume or diameter, and so was not available at a given threshold. The upper x-axis shows the number of features available for each threshold after the removal of volume-correlated features was performed. The feature number provided on the left y-axis can be cross-referenced with Table S1 to retrieve the given feature's name.

Table S3. Comparison of the normalized feature importance score and rank for all features with an importance score ≥0.75 for either correlation threshold = 1 (no features removed) or threshold = 0.25 (volume-correlated features removed). Shaded rows indicate features that were not highly important at threshold = 1, but became highly important at threshold = 0.25. Clinical features are underlined only to differentiate them more clearly against the radiomic features. The feature look-up number is provided to cross-reference this table with Figure S2, in which these look-up numbers correspond to the indices along the y-axis. Lastly, it should be noted that rows in this table are sorted according to the feature importance score/rank for correlation threshold = 0.25, and since the relative importance of the features changes at each threshold, the features are not in sorted order with respect to the threshold = 1 importance scores.

<table>
<tr><th rowspan="2">Feature Look-up Number</th><th rowspan="2">Feature Type</th><th rowspan="2">Feature Name</th><th colspan="2">Correlation Threshold = 1
(No Feature Removal)</th><th colspan="2">Correlation Threshold = 0.25</th></tr>
<tr><th>Importance Score</th><th>Importance Rank</th><th>Importance Score</th><th>Importance Rank</th></tr>
<tr><td>6</td><td>Radiomic – First Order</td><td>Kurtosis</td><td>0.878</td><td>10</td><td>1.000</td><td>1</td></tr>
<tr><td>48</td><td>Radiomic – GLCM</td><td>Inverse Variance</td><td>0.908</td><td>6</td><td>0.999</td><td>2</td></tr>
<tr><td>111</td><td>**Clinical**</td><td>**Primary Cancer Site**</td><td>0.990</td><td>2</td><td>0.992</td><td>3</td></tr>
<tr><td>112</td><td>**Clinical**</td><td>**Primary Cancer Histology**</td><td>0.926</td><td>4</td><td>0.972</td><td>4</td></tr>
<tr><td>106</td><td>Radiomic – NGTDM</td><td>Contrast</td><td>0.929</td><td>3</td><td>0.947</td><td>5</td></tr>
<tr><td>1</td><td>Radiomic – First Order</td><td>10th Percentile</td><td>0.811</td><td>15</td><td>0.825</td><td>6</td></tr>
<tr><td>35</td><td>Radiomic – GLCM</td><td>Cluster Shade</td><td>0.736</td><td>19</td><td>0.784</td><td>7</td></tr>
<tr><td>34</td><td>Radiomic – GLCM</td><td>Cluster Prominence</td><td>0.654</td><td>28</td><td>0.769</td><td>8</td></tr>
<tr><td>37</td><td>Radiomic – GLCM</td><td>Contrast</td><td>0.720</td><td>21</td><td>0.768</td><td>9</td></tr>
<tr><td>5</td><td>Radiomic – First Order</td><td>Interquartile Range</td><td>0.122</td><td>90</td><td>0.767</td><td>10</td></tr>
<tr><td>57</td><td>Radiomic – GLRLM</td><td>Gray-Level Non-Uniformity</td><td>1.000</td><td>1</td><td colspan="2" rowspan="12">*No feature importance score available since these features were removed due to being volume-correlated at this correlation threshold value*</td></tr>
<tr><td>45</td><td>Radiomic – GLCM</td><td>Inverse Difference Normalized</td><td>0.915</td><td>5</td></tr>
<tr><td>101</td><td>Radiomic – GLSZM</td><td>Zone Percentage</td><td>0.902</td><td>7</td></tr>
<tr><td>73</td><td>Radiomic – GLDM</td><td>Dependence Entropy</td><td>0.884</td><td>8</td></tr>
<tr><td>100</td><td>Radiomic – GLSZM</td><td>Zone Entropy</td><td>0.882</td><td>9</td></tr>
<tr><td>117</td><td>**Clinical**</td><td>**GTV Volume**</td><td>0.847</td><td>11</td></tr>
<tr><td>31</td><td>Radiomic – Shape & Size</td><td>Surface Volume Ratio</td><td>0.832</td><td>12</td></tr>
<tr><td>93</td><td>Radiomic – GLSZM</td><td>Large Area Low Gray-Level Emphasis</td><td>0.824</td><td>13</td></tr>
<tr><td>38</td><td>Radiomic – GLCM</td><td>Correlation</td><td>0.818</td><td>14</td></tr>
<tr><td>103</td><td>Radiomic – NGTDM</td><td>Busyness</td><td>0.803</td><td>16</td></tr>
<tr><td>58</td><td>Radiomic – GLRLM</td><td>Gray-Level Non-Uniformity Normalized</td><td>0.800</td><td>17</td></tr>
<tr><td>47</td><td>Radiomic – GLCM</td><td>Informational Measure of Correlation 2</td><td>0.795</td><td>18</td></tr>
</table>
